# Genome-wide identification of *Sclerotinia sclerotiorum* small RNAs and their endogenous targets

**DOI:** 10.1186/s12864-023-09686-7

**Published:** 2023-10-02

**Authors:** Roshan Regmi, Toby E. Newman, Yuphin Khentry, Lars G. Kamphuis, Mark C. Derbyshire

**Affiliations:** 1 Centre for Crop and Disease Management, School of Molecular and Life Sciences, Curtin University, Bentley, WA 6102 Australia; 2 Commonwealth Scientific and Industrial Research Organisation, Agriculture and Food, Floreat, WA 6014 Australia; 3 Present address: Microbiome for One Systems Health, CSIRO, Urrbrae, South Australia Australia

**Keywords:** Small RNAs, miRNAs, Gene regulation, Repeat elements

## Abstract

**Background:**

Several phytopathogens produce small non-coding RNAs of approximately 18–30 nucleotides (nt) which post-transcriptionally regulate gene expression. Commonly called small RNAs (sRNAs), these small molecules were also reported to be present in the necrotrophic pathogen *Sclerotinia sclerotiorum*. *S. sclerotiorum* causes diseases in more than 400 plant species, including the important oilseed crop *Brassica napus*. sRNAs can further be classified as microRNAs (miRNAs) and short interfering RNAs (siRNAs). Certain miRNAs can activate loci that produce further sRNAs; these secondary sRNA-producing loci are called ‘phased siRNA’ (PHAS) loci and have only been described in plants. To date, very few studies have characterized sRNAs and their endogenous targets in *S. sclerotiorum.*

**Results:**

We used Illumina sequencing to characterize sRNAs from fungal mycelial mats of *S. sclerotiorum* spread over *B. napus* leaves. In total, eight sRNA libraries were prepared from in vitro, 12 h post-inoculation (HPI), and 24 HPI mycelial mat samples. Cluster analysis identified 354 abundant sRNA clusters with reads of more than 100 Reads Per Million (RPM). Differential expression analysis revealed upregulation of 34 and 57 loci at 12 and 24 HPI, respectively, in comparison to in vitro samples. Among these, 25 loci were commonly upregulated. Altogether, 343 endogenous targets were identified from the major RNAs of 25 loci. Almost 88% of these targets were annotated as repeat element genes, while the remaining targets were non-repeat element genes. Fungal degradome reads confirmed cleavage of two transposable elements by one upregulated sRNA. Altogether, 24 milRNA loci were predicted with both mature and milRNA* (star) sequences; these are both criteria associated previously with experimentally verified miRNAs. Degradome sequencing data confirmed the cleavage of 14 targets. These targets were related to repeat element genes, phosphate acetyltransferases, RNA-binding factor, and exchange factor. A PHAS gene prediction tool identified 26 possible phased interfering loci with 147 phasiRNAs from the *S. sclerotiorum* genome, suggesting this pathogen might produce sRNAs that function similarly to miRNAs in higher eukaryotes.

**Conclusions:**

Our results provide new insights into sRNA populations and add a new resource for the study of sRNAs in *S. sclerotiorum.*

**Supplementary Information:**

The online version contains supplementary material available at 10.1186/s12864-023-09686-7.

## Background  

There is a complex interaction between pathogens and their hosts during infection [1]. These interactions can be studied with the patterns of gene expression and regulation obtained through RNA sequencing of both host and pathogen [2–5]. Although RNA sequencing helps to identify the key protein-coding genes involved in disease development, the complete picture of gene regulation also demands the investigation of sRNAs, which are 20–30 nucleotide non-coding RNA sequences that regulate gene expression in various biological processes including development and growth, maintenance of genome integrity, and responses to biotic and abiotic stress [6–8]. sRNAs regulate their target transcripts via sequence complementarity mediated by a process called RNA interference (RNAi) [9]. sRNAs are produced through the activity of the enzyme Dicer and are loaded into the RNA induced-silencing complex (RISC). The central protein is Argonaute. sRNAs then direct the complex to complementary nucleotide sequences, which can be mRNAs or DNA. Binding of the sRNA to DNA can lead to silencing of neighbouring genes through methylation while binding to an mRNA can lead to either translational repression or mRNA cleavage [10, 11].

After the discovery of RNAi in the fungus *Neurospora crassa* [12], numerous sRNA studies have been conducted in pathogenic and non-pathogenic fungal species, including *Cryptococcus neoformans* [13], *Trichoderma reesei* [14], *Puccinia triticina* [15], *Aspergillus flavus* [16], *Metarhizium anisopliae* [17], *Rhizophagus irregularis* [18], *Zymoseptoria tritici* [19], *Puccinia striiformis* [20], *Fusarium oxysporum* [21] and *F. graminearum* [22, 23]. Some species like *Ustilago maydis* and *Saccharomyces cerevisiae* have lost their RNAi capability [24–26], whilst other species of the same genera, such as *S. castellii* and *U. hordei,* have not, suggesting RNAi-related genes are not always essential [27, 28]. The endogenous roles of sRNAs in growth and development of filamentous fungi have been reported before in various studies [29–32].

There are two major classes of small RNA, including micro RNAs (miRNAs) and small interfering RNAs (siRNAs). Mature miRNAs are generally 20–24 nt sequences that originate from single-stranded RNA precursors with the ability to form hairpin structures with imperfectly paired arms, whereas siRNAs are formed from perfectly matched dsRNA precursors [33]. Phased siRNAs (phasiRNAs) are a secondary class of siRNAs produced via the miRNA-mediated cleavage of mRNAs or non-coding RNA precursors. They are generated at precise 21–22 nucleotide intervals from the miRNA cleavage site. Although there has been extensive research on phasiRNAs in plants [34–37], whether these are associated with fungal sRNAs has not yet been considered in detail. A single study has identified phasiRNAs in *S. sclerotiorum* during mycovirus infection [38]. However, no reports are available on whether phasiRNAs are expressed during infection.

In recent years, next generation sequencing has been used for the identification and investigation of fungal sRNAs [29, 39–41]. The fungus *S. sclerotiorum* is a pathogen of hundreds of plant species, including many crops, such as the economically important oilseed *Brassica napus* [42]. A few studies have been conducted on sRNAs in *S. sclerotiorum* [29, 43, 44]*.* In addition to this, changes in the *S. sclerotiorum* sRNA transcriptome were studied during mycovirus infection [45]. The first study conducted on sRNAs on *S. sclerotiorum* by Zhou et al. [43] identified 44 miRNA-like RNAs (milRNAs). This study was conducted only to identify the presence of miRNA-like structures in *S. sclerotiorum*. Later, Derbyshire et al. [44] identified 374 highly abundant *S. sclerotiorum* sRNAs during infection of two hosts, *Arabidopsis thaliana* and *Phaseolus vulgaris*. This study did not investigate any miRNA structures. Recently, Zihao et al. predicted 275 milRNAs associated with sclerotial development [29] and reported endogenous regulation of a histone acetyltransferase gene. Mochama et al. demonstrated the presence of antiviral RNA silencing mechanisms in *S. sclerotiorum* during mycovirus infection [45]. However, there are currently no sRNA transcriptome studies that have investigated the interaction with *B. napus*. It is worth investigating *S. sclerotinia* sRNAs with *B. napus* as a host, as Sclerotinia stem rot is a major yield-limiting factor for canola (*B. napus*) growers [42] and understanding molecular mechanisms at the sRNA level might provide some valuable resources for the management of SSR in canola.

With the goal of identifying endogenous *S. sclerotiorum* sRNA targets regulated in vitro and during infection, we sequenced sRNAs from liquid *S. sclerotiorum* cultures and mats of fungal mycelium lifted from infected *B. napus* tissue. We assessed whether any sRNA loci were differentially expressed at 12 and 24 HPI and computationally predicted targets of the sRNAs they produced, verifying 14 with degradome sequencing. Furthermore, we predicted miRNA and phasiRNA-producing (PHAS) loci; the latter of which have not been studied in detail in fungi to date. Together, the results from this study add a new resource for the study of sRNAs in *S. sclerotiorum.*

## Results

### Sequencing data analysis

**Fig. 1:**
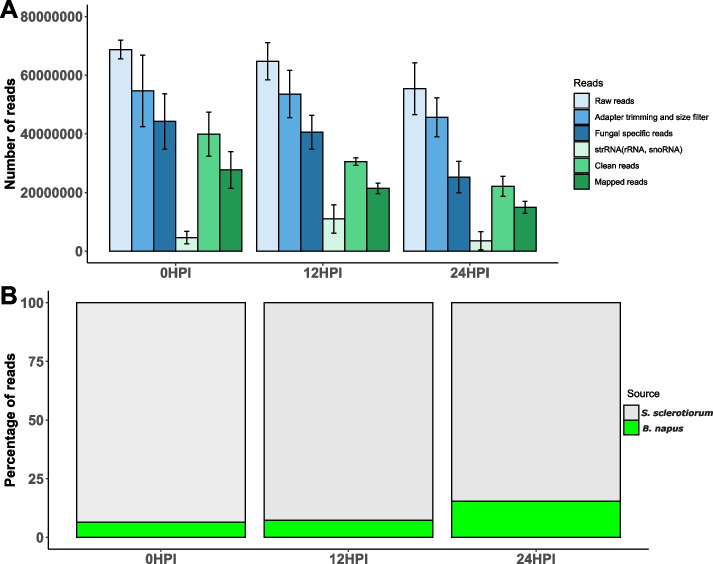
Analysis of small RNA sequencing datasets of *Sclerotinia sclerotiorum*. The x-axis shows the sRNA libraries from where the sRNA reads were generated, y-axis shows the average number of total reads identified for different reads from replicate samples for 0 HPI and 24 HPI while duplicates for 12 HPI. The error bars indicate standard deviation of the samples (**A**). Percentage of reads mapping to the *S. sclerotiorum* and *B. napus* genomes. The x-axis shows the replicates of treatment, and the y-axis shows the percentage of reads mapping to either *S. sclerotiorum* (grey) or *B. napus* (green)

We generated ~ 62 million high-quality raw reads from eight different *S. sclerotiorum* mycelial mat libraries. These were generated by growing *S. sclerotiorum* on minimal media and immediately harvesting the mycelial mat (0 HPI, in vitro sample) or transferring them onto *B. napus* leaves and harvesting the mycelial mats 12 and 24 HPI. Quality and size filtering resulted in a total of ~ 50 million reads. Among these reads, 72% mapped unambiguously to the *S. sclerotiorum* genome. The structural RNA filtering resulted in ~ 30 million clean fungal sRNA reads, which were used for prediction of sRNA / miRNA loci. Overall, ~ 69% of total sRNA reads were aligned to the fungal genome. Figure 1A shows a bar diagram summarising the sRNA sequencing dataset.

Surprisingly, an average of 6.5% of reads from uninoculated mycelial mats were best matched to the *B. napus* genome (Fig. 1B). Although there was no cross contamination of plant tissue in these samples such mapping of fungal originated sRNAs might be due to transposable element sRNAs that are common in the plant and fungus.

### Assessment of sRNA population and characteristics

**Fig. 2:**
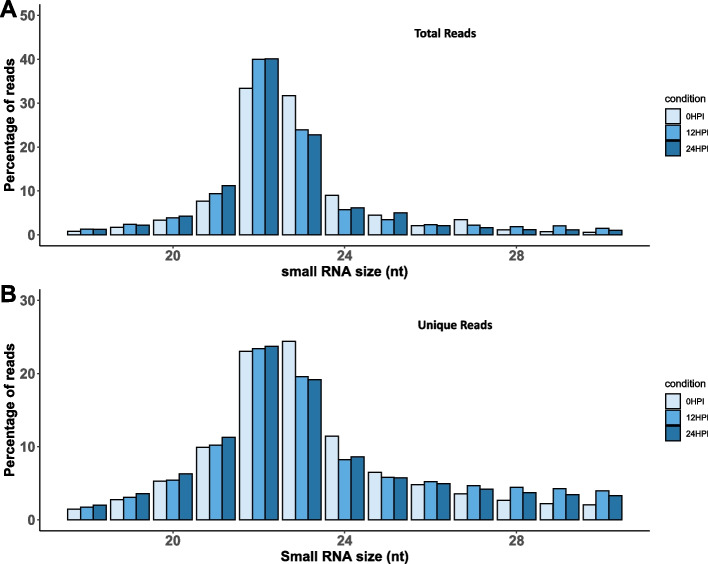
Length distribution of *Sclerotinia sclerotiorum* sRNAs. The percentage of reads (y-axis) according to nucleotide (nt) sequence length (x-axis) obtained in 0 HPI, 12 HPI, and 24 HPI pooled samples. Bar diagram of total reads (**A**) and unique reads (**B**)

**Fig. 3:**
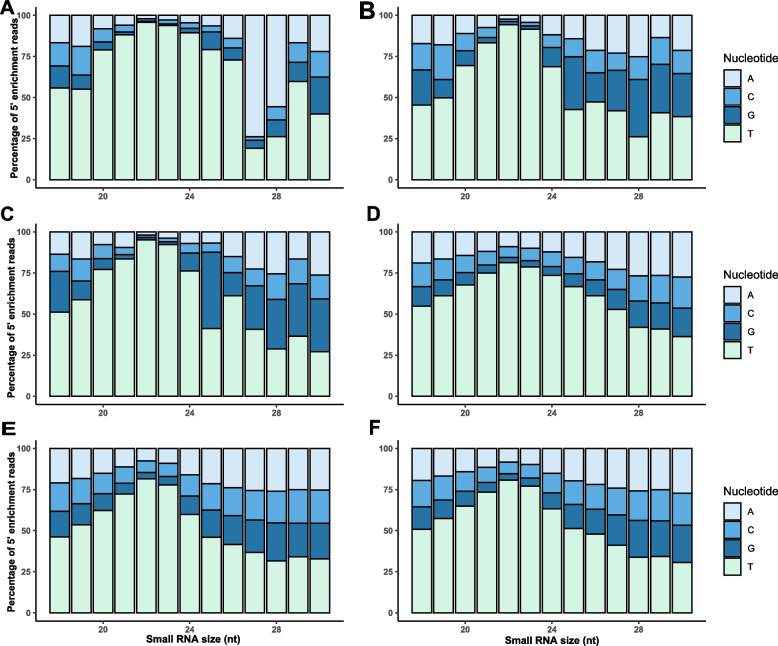
Nucleotide bias of *Sclerotinia sclerotiorum* small RNAs. The percentage of adenine (grey), cytosine (light blue), guanine (dark blue) and uridine (light green) in the 5’ nucleotide (nt) position according to read length for total reads (A-0HPI, B-12 HPI and C-24 HPI) and unique reads (D- 0HPI, E-12HPI and F-24HPI) of each sampling points

To understand the characteristics of the *S. sclerotiorum* sRNA population under different conditions, we examined the length distribution and 5’ nucleotide bias of sRNA libraries. Non-specific RNA degradation results in a uniform distribution of short RNA sequences, whereas Dicer-dependent generation of sRNAs results in a peak between 20 and 25 nt [33, 46, 47]. The distribution of sRNA sequences followed a typical sRNA size distribution, with the majority of reads between 20 and 24 nt long. The most frequent read length was 22 nt, followed by 23 nt for both total (Fig. 2A) and unique reads (Fig. 2B) in all samples. Highly abundant sRNAs in *S. sclerotiorum* had a bias toward uridine as the 5’ nucleotide. This characteristic is attributed to the sorting of sRNAs into Argonaute proteins [48]. Interestingly, at 0 HPI, total reads had a 5’ nucleotide bias of adenine at a length of 27 nt (Fig. 3). Previous studies have reported that *S. sclerotiorum* sRNAs exhibit a characteristic length distribution with most reads 22 and 23 nt in size with an enrichment of uracil as the 5’ nucleotide [46, 49]. Therefore, most of these sRNAs are processed through loading into Argonaute 1 proteins [40]. Assessing the abundance of both redundant and unique sRNA sequencing reads allows us to determine the complexity of the sRNAs expressed [50]. For example, if there are proportionally more redundant reads of a particular size than there are unique reads, it may indicate that sRNAs of that size are dominated by high levels of expression of a few unique sequences. In *S. sclerotiorum*, we found that unique and redundant reads had proportionally similar sizes, indicating a complex sRNA composition.

### Annotation of sRNA generating loci in *Sclerotinia sclerotiorum*

**Fig. 4:**
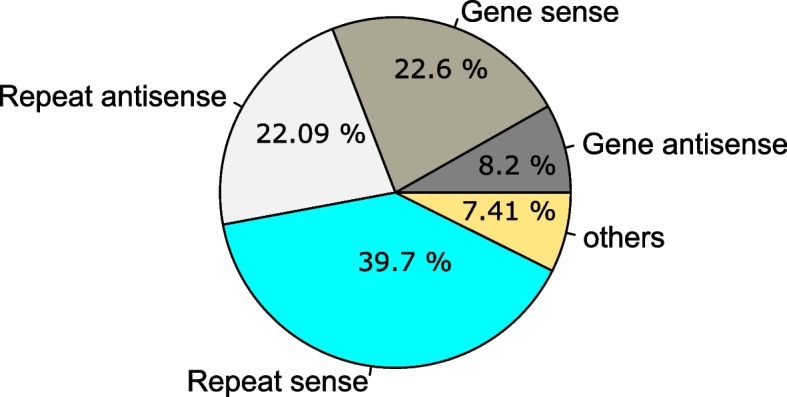
Assessment of sRNA origin of 1,073 Dicer-derived loci in the *Sclerotinia sclerotiorum* genome

ShortStack identified 1,073 Dicer-derived sRNA loci with 475 loci belonging to the negative strand of the chromosome and 598 from the positive strand (Supplementary Table 1). Among Dicer-derived loci, 245 originated from sense genic, 92 from antisense genic, 324 from repeat sense, and 670 from repeat antisense regions; a total of 354 loci had a read count of  >= 100 RPM. We further investigated the origins of these loci and found almost 62% overlapped with repeat elements, with 30% genes, and the remaining with other regions including intergenic and unannotated regions (Fig. 4).

### Identification of *Sclerotinia sclerotiorum* milRNAs

**Fig. 5:**
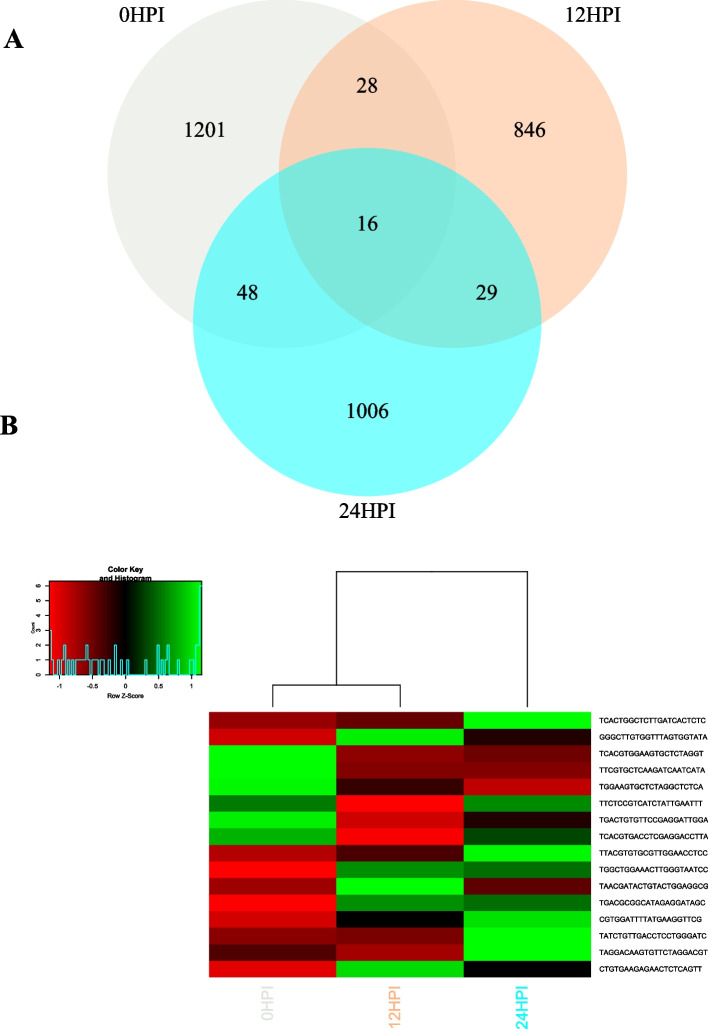
Venn diagram showing the number of milRNAs identified from *Sclerotinia sclerotiorum* from different libraries (**A**) and the heatmap showing the expression profile of 16 common miRNAs in three different libraries. X axis shows the pooled samples from each time points and Y-axis shows the mature milRNA sequences, the color key indicates Raw z-score of normalized counts in Reads Per Million (**B**)

To identify whether *S. sclerotiorum* encodes milRNAs, we employed the miRCat tool implemented in UEA sRNA Workbench using default parameters [51]. It is also worth mentioning here that running ShortStack in -hp mode also predicts miRNA loci. The ShortStack program did not predict any miRNA loci from virus-infected *S. sclerotiorum* in a previous study [52]. Here, we identified only one milRNA locus using the ShortStack program. The original location of this locus was Scaffold_74:155–245 with an annotated “UAAAGCGCAUCACUAGUAAUUCUUCGUGUUACUACCUAUCUCCAUCUGAAUGGGUAGAAGCACGAAUGGAUGAUGAUGAGAUCCUAGGUUC and a mature sequence of UGAAUGGGUAGAAGCACGAAUGGAU” (Supplementary Fig. 1) hairpin structure. In total, 30,849 sequence reads were found for this miRNA. Interestingly, a homology search of this miRNA sequence identified four closely matched miRNAs deposited in miRBase [53], including egr-miR-10241-5p [54], mdo-miR-7398l-3p [55], mdo-miR-7398-5p [55] and oni-miR-10798 [56]. In contrast, miRCat predicted a greater number of milRNA loci. Altogether, 1,313, 944, and 1,139 miRNA loci were predicted from 0, 12, and 24 HPI libraries, respectively, and these contained 1,293, 919, and 1,099 non-redundant miRNA sequences, respectively. However, only 44 and 64 miRNA sequences were common to 0 HPI + 12 HPI and 0 HPI + 24 HPI libraries, respectively. Furthermore, 16 sequences were common to all libraries (Fig. 5A). Expression profile of these sequences across three time points is shown in Fig. 5B. From our dataset, we found 24 milRNA loci with both mature and miRNA* sequences, and these contained 20 unique milRNAs. We found homology for 12 miRNA sequences in the miRBase database. Details of these loci are shown in Table 1

**Table 1:** Description of 23 miRNA loci with mature miRNA and miRNA* sequences with homology to known miRNA sequences in miRBase identified in the *Sclerotinia sclerotiorum* CU8.24 genome using miRcat software

MilRNA loci	milRNA	Abundance	Homology	Organism	Samples
scaffold_117: 55,559–55,578	AATACGCGCTGCCGAGAATC	16	sja-miR-3482-3p	*Schistosoma japonicum*	12HPI
scaffold_184: 22,040–22068	TCGGACTCGGCGTAATCTGCG	135	ata-miR166b-5p	*Aegilops tauschii*	12HPI
scaffold_74: 205–226	TTCTTCGTGTTACTACCTATCT	11,885	NA		12HPI
scaffold_169: 52,589–52,610	TATATAGATCTTCGTAACTAGG	3970	NA		24HPI
scaffold_206: 1453–1474	TCACTGGCTCTTGATCACTCTC	62,437	bmo-miR-3260, ppc-miR-8256-5p, tca-miR-3872-5p	*Bombyx mori, Pristionchus pacificus, Tribolium castaneum*	24HPI
scaffold_222: 10,335–10356	TAAGGCGGGAACATTTTTGAGG	77	cpo-miR-507a-3p	*Cavia porcellus*	24HPI
scaffold_286: 12,367–12,386	ACCGAATCTGGGTTTGAGAC	16	ptc-miR6459b, ptc-miR6459a-3p	*Populus trichocarpa*	24HPI
scaffold_32: 79,430–79451	TCACTGGCTCTTGATCACTCTC	62,437	bmo-miR-3260, ppc-miR-8256-5p, tca-miR-3872-5p	*Bombyx mori, Pristionchus pacificus, Tribolium castaneum*	24HPI
scaffold_337: 10,265–10286	TCACTGGCTCTTGATCACTCTC	62,437	bmo-miR-3260, ppc-miR-8256-5p, tca-miR-3872-5p	*Bombyx mori, Pristionchus pacificus, Tribolium castaneum*	24HPI
scaffold_10: 47,497–47,518	TCGTGCTTGATGTCTTTGAGGT	4	gma-miR862a, gma-miR862b	*Glycine max*	0HPI
scaffold_133: 52,648–52,669	TTTAGGTCTAGGTCGTCTGTCT	18	NA		0HPI
scaffold_165: 31,316–31,337	TGTGCGGCTGTGGGACTAAAGC	58	NA		0HPI
scaffold_169: 52,589–52,610	TATATAGATCTTCGTAACTAGG	11,616	NA		0HPI
scaffold_18: 286,294–286,315	TTTCCCTGAGGTATCCTGGCTA	37	crm-miR-64d-3p	*Caenorhabditis remanei*	0HPI
scaffold_19: 246,184–346,203	TCGATTTGAGATAGTGTCTC	100	NA		0HPI
scaffold_194: 11,136–11,157	TAGCACTTCTAGGGATTCCGCC	24	NA		0HPI
scaffold_218: 18,029–18049	TCATGAAGGGTTATGGTGGGT	30	aca-miR-5449	*Anolis carolinensis*	0HPI
scaffold_319: 6400–6421	TGTGTGTGTATGATTCTATTGC	40	oni-miR10680, mmu-miR-1187, atr-miR8565g	*Oreochromis niloticus, Mus musculus, Amborella trichopoda*	0HPI
scaffold_52: 64,586–64,607	TCTCAAAGGATCTGTTCAGCAC	112	vca-miR10208-5p, egr-miR-10276-3p	*Vriesea carinata, Echinococcus granulosus*	0HPI
scaffold_56: 5103–5124	TAGTAGTGACTCTTCCTCGGAT	45	gga-miR-6559-5p	*Gallus gallus*	0HPI
scaffold_7: 13,512–13,533	TGTTACGCTGCGGAATTTGACA	46	gga-miR-6579-5p	*Gallus gallus*	0HPI
scaffold_71: 61,583–61,604	TGACTGTGTTCCGAGGATTGGA	126	ath-miR2111b-3p	*Arabidopsis thaliana*	0HPI
scaffold_74: 204–225	TCTTCGTGTTACTACCTATCTC	17,753	NA		0HPI

### Small RNAs are differentially expressed in *Sclerotinia sclerotiorum* mycelium after host inoculation.

**Fig. 6:**
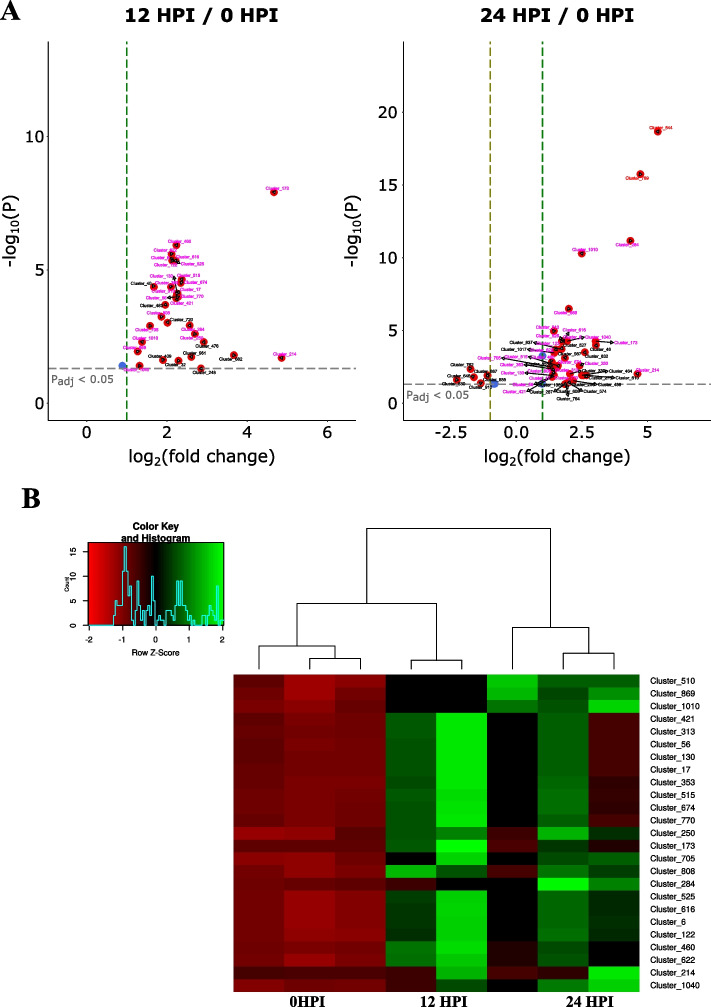
Analysis of differentially expressed small RNA loci/clusters. (**A**) Volcano plots of differentially expressed small RNA clusters in 12 HPI and 24 HPI relative to 0 HPI (in vitro) plotted as log_2_ fold change as a function of -log_10_ p adj value calculated from a Wald p-test in DeSeq2. Cluster 510 was not shown in the volcano plot for 12 HPI as the log_2_ fold change was ~ 0.9. Clusters in pink are upregulated at both 12 and 24 HPI time points relative to 0 HPI, clusters in red (Cluster_544 and Cluster_789) are significantly upregulated at 24 HPI relative to 12 HPI. The blue circle and horizontal broken line represent padj cut off value of 0.05 while the vertical broken lines represent log_2_ fold cut off values of -1 and 1. (**B**) Heatmap of normalized expression data from the 25 ShortStack loci commonly up regulated across both time points, 12 and 24 h post inoculation (HPI)

The individual raw reads for each library from ShortStack were normalized using DESeq2 in R [57]. We used the 354 abundant loci with sRNA reads of more than 100 RPM to find differentially expressed clusters. Altogether, 34 and 57 clusters were altered in their expression profiles, with adjusted p-value of less than 0.05, across 12 and 24 HPI time points, respectively (Fig. 6A). From these loci, 25 clusters were upregulated at both timepoints post infection (Fig. 6B), suggesting they were infection-induced.

### Prediction of phased loci in *Sclerotinia sclerotiorum*

To identify phasiRNAs in *S. sclerotiorum* we used the UEA sRNA Workbench [51]. Altogether, 26 phased loci were predicted from combined in vitro and infected samples. In total, 217 and 147 unique phasiRNAs were produced from these loci. Detection of loci producing phasiRNAs indicates that some *S. sclerotiorum* sRNAs could act in a similar fashion to miRNAs in higher organisms [52]. Details of predicted phased loci are shown in Supplementary Table 2.

### Endogenous targets of fungal sRNAs were mostly repetitive elements

**Fig. 7:**
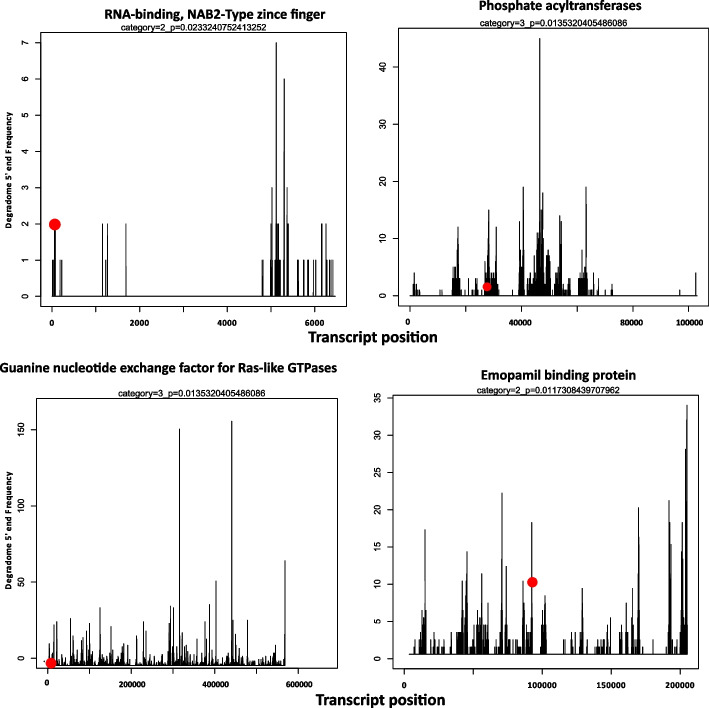
The target plots of representative four targets with known domains showing the distribution of the degradome tags along the full length of the target sequence in CU8.24 genome. The red circle represents the sliced target transcripts

For target prediction, we divided *S. sclerotiorum* sRNAs into two sets. The first consisted of 25 major sRNAs from loci up-regulated commonly across both 12 and 24 HPI timepoints and the second comprised 165 milRNAs, predicted using miRCat, with > = 100 reads found in all libraries. From the psRNA online target prediction tool, with a maximum expectation score of 3.5 [58], 343 targets were predicted in the *S. sclerotiorum* genome from 25 upregulated sRNAs. Among these targets, 54 were annotated as genes while 343 targets overlapped with repeat elements. We also used our fungal specific degradome sets to see whether there was any evidence of cleavage of endogenous targets. Interestingly, we found two transposable element targets from the analysis of the degradome dataset (Supplementary Fig. 2). Furthermore, we found 4,917 endogenous targets regulated by 165 milRNAs. Among these targets, 475 were annotated as genes, while 4,621 were related to repeat elements. Furthermore, we found evidence for cleavage of 12 targets from our fungal specific degradome dataset. One of the targets “sscle_01g003340” was predicted to be cleaved by four milRNAs which encodes a domain related to RNA-binding, a NAB2 type zinc finger. Other confirmed targets were “sscle_03g028020’’, “sscle_15g102960’’, “sscle_03g030160”, “sscle_07g059010*’’*, which encode proteins with domains related to phosphate acyltransferases, eukaryotic protein of unknown function (DUF829), emopamil binding protein, guanine nucleotide exchange factor for Ras-like GTPases; N-terminal motif, respectively. The remaining four targets were “sscle_15g106630’’, “sscle_15g106740’’, “sscle_08g066610*”,* “sscle_01g011410*”* which had no known Pfam domains. The representative target plots of four identified targets with annotated Pfam domains is shown in Fig. 7.

## Discussion

Small RNAs are short non-coding RNAs derived from either from double-stranded or hairpin RNAs [13, 35]. Depending on the precursor and biogenesis pathways, sRNAs can be classified as siRNAs and miRNAs. siRNAs are formed from double-stranded precursors while miRNAs are formed from hairpin loop structures [10]. Besides this, there are other categories of siRNAs identified in plants and animals like hairpin-siRNAs, natural-antisense siRNAs, heterochromatic siRNAs, and secondary siRNAs. PhasiRNAs are a subclass of siRNA which are produced by miRNA-mediated cleavage of mRNAs or non-coding RNAs [59]. In our study, we identified 1,073 Dicer-derived loci, 165 highly expressed milRNA loci, and 26 phased loci in the *S. sclerotiorum* genome that were identified from sRNA sequencing datasets from in vitro cultures and infected detached leaves.

In this study, *S. sclerotiorum* mycelial mats sampled from three different stages were used to develop sRNA libraries. The size distribution of total and unique reads was investigated. The length distribution of sRNAs in *S. sclerotiorum* peaked at 22 and 23 nt, which is fairly similar to other fungi like *F. oxysporum* [31], *P. marneffei* [60] and *A. flavus* [16]. Previous studies in *S. sclerotiorum* report that the most abundant sRNAs are 22 and 23 nt in length with a 5’ bias of uridine [29, 44], which agrees with the findings of this study with 5’ bias of uridine. The former study identified 374 highly abundant sRNAs during infection of two host plants *A. thaliana* and beans while the latter study has analysed the response of *S. sclerotiorum* during mycovirus infection. We found a 5’ uridine bias in the most abundant sRNAs, suggesting that these sRNAs bind to AGO1 [48]. Interestingly, total reads of 0 HPI had a 5’ nucleotide bias of adenine at a length of 27 nt. While there are no reports of such a bias in fungal sRNAs, 5’ adenine biased siRNAs have been previously reported to be involved in RNA dependent DNA methylation in *A. thaliana*, mediated by the Argonaute protein AGO4 [48].

sRNA mediated RNAi is an important gene regulation mechanism in plants, animals and fungi [11]. milRNAs in fungi, similar to those of plants and animals, have recently been identified to regulate different processes like development, pathogenesis and reproduction [29, 31, 61]. It has been well demonstrated that sRNAs play key roles in development and stress responses. In this study, we identified 25 commonly upregulated sRNAs at 12 and 24 HPI during *B. napus* infection. The expression patterns of these sRNAs suggest possible roles in growth and pathogenicity on *B. napus*. sRNAs have previously been shown to have a key role in regulation of transposable elements [15, 62].

Target identification of sRNAs including milRNAs revealed almost 90% of the targets as repeat elements. sRNAs contribute to maintaining genome integrity by regulation of repeat elements. It is not surprising to see regulation of repetitive elements by fungal sRNAs, since one of the major functions of sRNAs is silencing of repeats/transposons to maintain genome integrity [40]. In the wheat pathogen *P. triticina*, 32.19% of endogenous targets of pathogen sRNAs were repeat elements [15]. This research further enhances our knowledge of sRNA classes in *S. sclerotiorum* and how they change over time during the interaction with *B. napus*.

Although there were hundreds of targets identified from in silico predictions (343 from 25 major upregulated sRNAs and 4917 from 165 highly expressed milRNAs), we were able to confirm the endogenous cleavage of 14 targets from this study using degradome sequencing data. Interestingly, domains related to binding factor, exchange factor and phosphate acetyltransferase were encoded by these genes. Fungal pathogen-induced phosphate acetyltransferase genes have been reported to be involved in host-pathogen interactions by affecting the host cell wall and causing dehydration of leaves [63, 64]. Also, acetyltransferase genes have been reported to have a role in sclerotial and conidia development in *S. sclerotiorum* and *Pestalotiopsis microspora* [29, 65]. Our finding further echoes that endogenous gene regulation by sRNAs has a potential role in the pathogenicity of *S. sclerotiorum.*

Interestingly, the gene model sscle_03g028020 was cleaved by four milRNAs suggesting *S. sclerotiorum* milRNAs endogenously regulate this class of genes. RNA-binding, NAB2-type zinc finger domains are involved in poly (A) tailing of mRNAs [66]. The way a mRNA binds to RNA-binding proteins determines its fate for further functions. Poly(A) tailing is especially important for regulation of transcript stability [67]. The gene model sscle_01g003340 identified here has a domain related to RNA-binding proteins, therefore fungal milRNAs may also play an important role in transcript stability. The emopamil binding protein is a membrane protein of the endoplasmic reticulum. In *B. cinerea*, this protein has a role in virulence [68]. The gene model sscle_03g030160, which encodes an emopamil binding protein in *S. sclerotiorum*, is regulated by milRNAs, suggesting milRNAs could play a role in the virulence of *S. sclerotiorum*. Guanine nucleotide exchange factor for Ras-like GTPases; N-terminal motif are involved in the catalytic activity of exchange factors in fungi [69, 70]. These classes of genes have been shown to be involved in vesicle trafficking, endocytosis and development processes in *F. graminearum* [70]. The gene model sscle_07g059010 identified here to be regulated by milRNAs may be important for similar processes in *S. sclerotiorum*.

Fungi are one of the three lineages of eukaryotes, along with plants and animals [71]. Although the sRNA machinery is present in fungi, plants and animals, conserved miRNAs are often not detected. So far, no homologs of *S. sclerotiorum* miRNAs have been identified in plants and animals. In this study, we identified 13 miRNAs which have homologues in other kingdoms. This result indicated that some miRNAs might be conserved between kingdoms [72, 73]. These identified milRNA homologues were reported to have a role in morphology, bi-directional movement, nutrient metabolism and in embryonic development in worms and mammals [54, 56].

We also showed that *S. sclerotiorum* might regulate gene expression through the production of secondary siRNAs like phasiRNAs. In plants, phasiRNAs are either produced by mRNAs or non-coding RNAs [74]. The loci producing phasiRNAs are called PHAS genes. Endogenous phasiRNAs play important regulatory roles by silencing protein coding transcripts and are associated with pathogen infection [75]. Here, we identified 26 PHAS genes with 147 phasiRNAs with 730 targets in *S. sclerotiorum* using psRNA Target server with available *S. sclerotiorum* transcripts in the server (Supplementary Table 3). Among these targets, 207 belong to the expectation score of 0 suggesting these are high confidence targets. Most of these are either hypothetical or predicted proteins. In summary, this study enhances the knowledge of endogenous *S. sclerotiorum* sRNAs and their targets.

## Methods

### Fungal and plant sources

The sclerotia of *S. sclerotiorum* isolate CU8.24 collected from South Stirling in the Western Australia by Denton-Giles et al. [76] was used to generate sRNA dataset from in vitro and infected *B. napus* leaves in this study. The *B. napus* seeds (variety AV-Garnet) were originally acquired from The Australian Grains GeneBank (accession AGG95718BRAS1).

Fully mature, dry sclerotia were cut in half and placed mycelium-side down on potato dextrose agar (PDA) (Becton Dickinson, USA) and incubated for a week at 20 ºC in the dark. The germinated mycelium was subcultured onto fresh PDA. After 48 h of incubation, a 1 cm mycelial plug was inoculated onto minimal medium, which consists of 2 g/L NH_4_NO_3_, 1 g/L KH_2_PO_4_, 0.1 g/L MgSO_4_.7H_2_O, 0.5 g/L yeast extract, 4 g/L DL-malic acid and 1 g/L NaOH [77]. The two-day old mycelial mat from the minimal medium was spread over a detached first leaf of one-month-old *B. napus* (cv AV Garnet), placed in a Petri dish containing wet filter paper and incubated at 20 °C. After 12 and 24 HPI, a symptomatic lesion was observed on the surface of the leaves and intact mycelium was carefully separated from the leaves and immediately frozen in liquid nitrogen and stored at -80 °C [78] until RNA extraction. The in vitro mycelial mat before inoculation (0 HPI) was used as a reference to investigate sRNA transcriptome changes.

### RNA isolation and sequencing

*S. sclerotiorum* mycelial mats were collected from *B. napus* detached leaves at 12, and 24 HPI. The fungal mycelium mat that has been collected immediately from liquid culture without putting on leaves was treated as 0 HPI.

The collected fungal material was ground into a fine powder with liquid nitrogen with a pre-cooled RNase-free mortar and pestle. Total RNA was extracted using the Trizol™ reagent following the manufacturer’s protocol (Invitrogen, Carlsbad, CA, USA). Total RNA was quantified using a NanoDrop spectrophotometer and Qubit (Invitrogen) and the integrity was checked with agarose gel electrophoresis. Eight libraries of sRNAs were prepared from total RNA from three replicates each for in vitro mycelium and 24 HPI and duplicates for 12 HPI using the TruSeq small RNA sample preparation kit (Illumina, San Diego, CA, USA) according to the manufacturer’s protocol. Thereafter, single-end (50 bp) sequencing was performed on an Illumina PE150 by Novogene (Novogene, Beijng, China). The raw reads generated in this study are available in the National Centre for Biotechnology Information Centre (NCBI) Sequence Read Archive (SRA) under BioProject PRJNA985401.

### Analysis of sequencing data

Total raw reads were trimmed using Cutadapt v1.15 [79], using the command ‘cutadapt -a adaptor.fa -o trimmed. fq raw.fq -m 18 -M30’, where ‘adaptor.fa’ contains the adaptor sequences used for the sRNA library preparation. Sequences of a length of 18–30 nt were retained, which is the typical size of sRNAs. The quality of filtered reads was assessed with FastQC v0.11.8 [80]. Trimmed reads were assigned to the genome of the *S. sclerotiorum* isolate CU8.24 [81] and the *Brassica napus* V.5 genome [82] using bbsplit [83] with the ‘ambig2’ option set to ‘toss’, which discards reads that map equally well to both host and pathogen. Reads that were unique to the fungal genome were kept for prediction of *S. sclerotiorum* sRNAs. The structural RNA RFAM hits were mapped against the CU8.24 genome to identify their location in the genome to filter out *S. sclerotiorum* specific sRNAs potentially derived from structural RNA (ribosomal RNA, snRNA, and snoRNA) using the Rfam database [84] and the program Infernal v1.1.3 with the command ‘cmscan –nohmmonly –rfam –cut_ga –fmt 2 –oclan –oskip -o strRNA.out –tblout strRNA.tblout structuralRNA.cm CU8_24.fasta’ [85]. At first, the Rfam hits of structural RNAs in the CU8.24 genome were prepared from the Rfam clanin file using above command. The strRNA.tboult output from first step was used to find structural RNA loci by using the command grep -v \^# strRNA.tblout | awk ‘{print ("%s/%d-%d %d %d %s\n", $1, $8, $9, $8, $9, $1);}' | esl-sfetch -Cf CU8.24.fasta—> strRNACU8.24.fasta. The annotated structural RNAs file strRNACU8.24. fasta was used as a reference file to map sRNAs originated from the structural RNA in CU.8.24 genome. using Bowtie2 [86]. Reads that were not aligned/mapped were used for prediction of sRNA loci. BEDTools v2.29.0 [87] was used to find reads overlapping to the genes and repetitive elements.

The clean reads of each library were used as a single entity to find sRNA-producing loci with the program ShortStack v3.8.5 (ShortStack –readfile $fastq_dir –genomefile $Genome –outdir $outdir –bowtie_cores 6 –sort_mem 10G –mismatches 0 –mmap f –bowtie_m all –mincov 100rpm –dicermin 18 –dicermax 30) [33]. ShortStack first identifies significant alignment coverage based on depth of alignment. Significant alignments are then expanded upstream and downstream. Overlapped regions are thereafter merged to form clusters [46]. Clusters are annotated as ‘Dicer-derived’ (Dicer being the main enzyme that generates mature sRNAs) loci when 80 percent of reads have a length of 18–30 nt. The most abundant sRNAs of Dicer-derived loci are referred to as ‘major RNAs’. Sequences with miRNA-like features were predicted using the miRCat tool implemented in the UEA sRNA Workbench v4.5 with default parameters [51]. For prediction of miRNAs, sRNA libraries for each treatment were pooled to make a single file, therefore, three miRCat runs were conducted for 0, 12 and 24 HPI. This program uses PatMan to map sRNA reads to the input genome, using a flank extension of 100 bp, a series of putative miRNA precursors are then excised. Afterward, secondary structures of these putative precursors are predicted and retained below a minimum free energy threshold -25 and *P*-value of 0.05 using RNAfold v.2 [88]. We also used a second approach to predict miRNA loci using ShortStack. We focused on miRNA loci with both miRNA and miRNA* sequences as the presence of the miRNA* sequence in the locus provides strong evidence that these miRNAs are Dicer-derived [43].

### Differential expression analysis

We used DESeq2 Bioconductor package in R 3.6.1 to normalize the total raw read counts from ShortStack for differential expression analysis [57]. After normalization of counts, we compared expression of sRNA loci/clusters between in vitro and 24 HPI, in *vitro* and 12 HPI, and 12 HPI and 24 HPI using the negative binomial test. Differentially expressed clusters were determined using Benjamin-Hochberg false discovery rate [89] to adjust *P* values ​​based on the Wald test. Clusters with a log-change ≥ 1 (or − 1 or less) and an adjusted *P* value < 0.05 were defined as differentially expressed clusters.

### Small RNA target prediction

Target prediction of sRNAs was done using the psRNATarget online tool (2017 release) [58]. All the parameters were kept default except the expectation score (< 3). The psRNATarget server finds target sequences based on complementarity according to a predefined scoring scheme and cleavage site recognition by calculating a threshold ratio of unpaired minimum free energy to paired minimum free energy [58]. We used two different approaches for endogenous target detection. The first approach simply comprised the input of identified sRNA datasets and *S. sclerotiorum* 1980 transcripts, National Center for Genetic Engineering and Biotechnology, available in psRNATarget server. This approach might miss some of the genuine targets and it does not give enough information on repeat element genes. In the second approach the sRNA datasets and the *S. sclerotiorum* CU8.24 genome were used as input files with the psRNA target tool. The sRNA/mRNA complementary sites were then used to find their positions in the *S. sclerotiorum* genome using BEDTools v2.29.0. We separately used genes and repeat elements files of CU8.24 previously annotated [81] to characterize the predicted targets as repeat elements and non-repeat element (e.g. genes or introns) with BEDTools. Any targets that overlapped with repeat elements were called repeat element genes and the remaining as non-repeat element genes. For the functional annotation of the targets, 200 bp upstream and downstream were excised and compared with *S. sclerotiorum* InterPro and Pfam domains.

### Detection of PHAS loci

The PHAS loci were predicted using ta-si prediction tool from UEA sRNA Workbench [51]. This software requires an sRNA dataset and a genome file for input. We separately ran the collapsed file for 0 HPI, 12 HPI and 24 HPI to find the PHAS loci across all time points. At first, the program aligns sRNAs to the genome using PATMAN and any sRNAs that are not matched to the genome are discarded. Using the algorithm developed by Chen et al. [90], it calculates the probability of the phasing being significant based on the hypergeometric distribution. Only 21 nt sRNAs were used in the phasing analysis.

### Degradome analysis

To find putative cleavage sites, we used fungal specific reads from degradome sequencing generated from infected *in planta* samples at 24 HPI from our previous study that was deposited in the SRA under BioProject PRJNA678586. Detail about the degradome analysis was mentioned in our previously published article [91]. In brief, construction of the degradome library was started from the degradation site (with a 5’ monophosphate group) of the degraded mRNA. The sequencing adaptors were added to both ends of the degraded mRNAs and a library with an insert size of around 200–400 (base pairs) bp was generated. Paired end sequencing (2 × 150bp) was performed on a Hiseq 2500.

### Supplementary Information

**Additional file 1: Supplementary Figure 1. **Secondary hairpin structure of milRNAloci predicted from ShortStack in Sclerotinia sclerotiorum genome. The intensity of colour signifies base pair possibilities.

**Additional file 2: Supplementary Figure 2.** Target plots of two transposable elements genes verified from the fungal specific degradome dataset. X-axis shows the transcript position while Y- axis shows the degradome frequency. Red circle indicates the cleavage point in the transcript. Category signifies the confidence of the targets.

**Additional file 3: Supplementary Table 1. **Detail description of small RNA loci predicted from Sclerotinia sclerotiorum genome CU8.24 using ShortStack program. **Supplementary Table 2.** Phased loci predicted in Sclerotinia sclerotiorum genome. **Supplementary Table 3.** Targets of phasiRNAs predicted in Sclerotinia sclerotiorum genome 1980 using psRNA target server

## Data Availability

The sRNA dataset generated and analysed during the current study are available in the NCBI SRA repository under BioProject PRJNA985401. The degradome data analysed during the current study are available in the NCBI SRA under BioProject PRJNA678586. *Brassica napus* material was obtained from the Australian Grains Genebank, accession AGG95718BRAS1 under a non-commercial standard MTA.

## Abbreviations

nt: Nucleotides
sRNA: Small RNA
miRNA: MicroRNA
siRNA: Short interfering RNA
PHAS: Phased short interfering RNA
HPI: Hours post-inoculation
RPM: Reads per million
milRNA: MicroRNA-like RNA
phasiRNA: Phased short interfering RNA
RNAi: RNA interference
RISC: RNA-induced silencing complex
dsRNA: Double-stranded RNA
