# Supplementary material for: Genome-wide identification of Sclerotinia sclerotiorum small RNAs and their endogenous targets
Source: BMC Genomics. 2023 Oct 2;24:582. doi: 10.1186/s12864-023-09686-7 (PMC10544508; doi:10.1186/s12864-023-09686-7)
Supplement: Supplementary file 1 — Additional file 1: Supplementary Figure 1. Secondary hairpin structure of milRNAloci predicted from ShortStack in Sclerotinia sclerotiorum genome. The intensity of colour signifies base pair possibilities. [file 12864_2023_9686_MOESM1_ESM.docx]

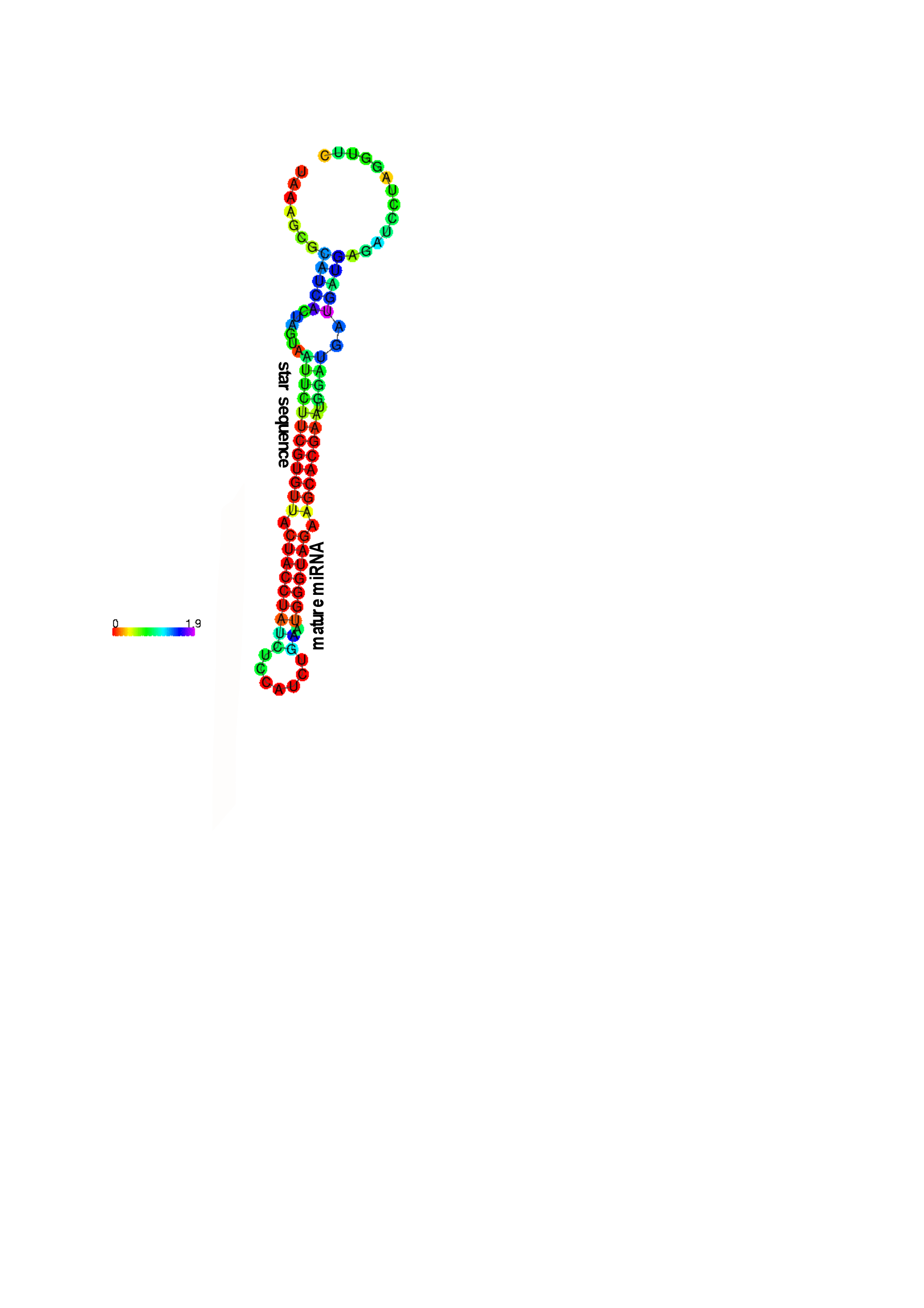


Supplementary figure 1 Secondary hairpin structure of milRNAloci predicted from ShortStack in Sclerotinia sclerotiorum genome. The intensity of colour signifies base pair possibilities.
