# Supplementary material for: Genome-wide identification of Sclerotinia sclerotiorum small RNAs and their endogenous targets
Source: BMC Genomics. 2023 Oct 2;24:582. doi: 10.1186/s12864-023-09686-7 (PMC10544508; doi:10.1186/s12864-023-09686-7)
Supplement: Supplementary file 2 — Additional file 2: Supplementary Figure 2. Target plots of two transposable elements genes verified from the fungal specific degradome dataset. X-axis shows the transcript position while Y- axis shows the degradome frequency. Red circle indicates the cleavage point in the transcript. Category signifies the confidence of the targets. [file 12864_2023_9686_MOESM2_ESM.docx]

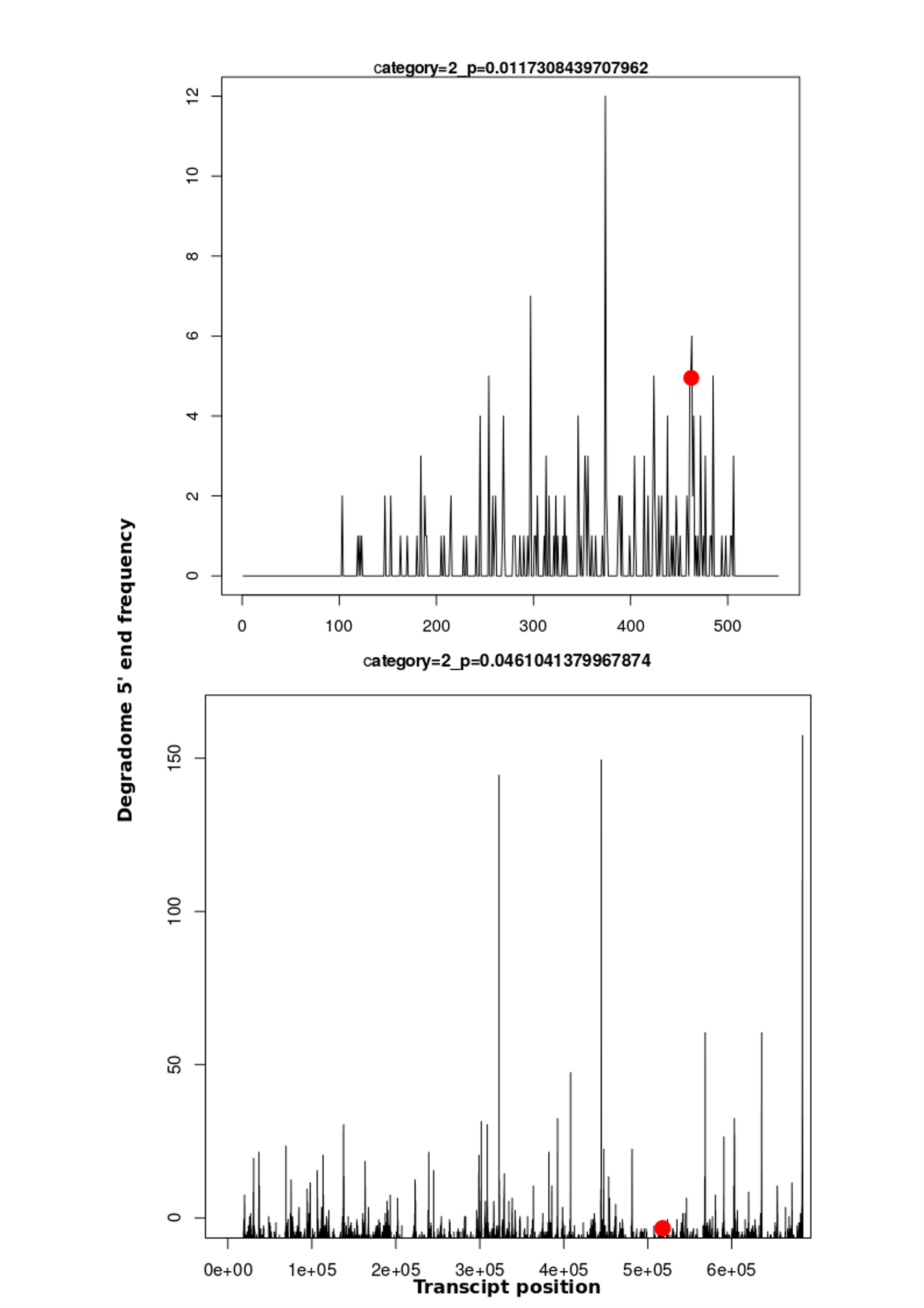


Supplementary Figure 2. Target plots of two transposable elements genes verified from the fungal specific degradome dataset. X-axis shows the transcript position while Y- axis shows the degradome frequency. Red circle indicates the cleavage point in the transcript. Category signifies the confidence of the targets.
